# Shifts in morphology, gene expression, and selection underlie web loss in Hawaiian *Tetragnatha* spiders

**DOI:** 10.1186/s12862-021-01779-9

**Published:** 2021-03-22

**Authors:** Cory A. Berger, Michael S. Brewer, Nobuaki Kono, Hiroyuki Nakamura, Kazuharu Arakawa, Susan R. Kennedy, Hannah M. Wood, Seira A. Adams, Rosemary G. Gillespie

**Affiliations:** 1 Department of Environmental Science, Policy and Management, University of California, Berkeley, 130 Mulford Hall, #3114, Berkeley, CA 94720-3114 USA; 2 Present Address: MIT-WHOI Joint Program in Oceanography/Applied Ocean Science and Engineering, Cambridge, Woods Hole, MA USA; 3 Department of Biology, N1088 Howell Science Complex, East Carolina University, Greenville, NC 27858 USA; 4 Institute for Advanced Biosciences, Keio University, Yamagata, Japan; 5 Enzyme Research Team, RIKEN Center for Sustainable Resource Science, 2-1 Hirosawa, Wako-shi, Saitama, 351-0198 Japan; 6 Biodiversity and Biocomplexity Unit, Okinawa Institute of Science and Technology Graduate University, Tancha 1919-1, Onna, Okinawa, 904-0495 Japan; 7 Smithsonian Institution, Entomology, MRC105, Natural History Bldg. E519, 1000 Constitution Ave NW, Washington DC, 20560-0188 USA

**Keywords:** Convergence, *Tetragnatha*, Spidroins, Web loss, Transcriptomics, Selection, Gene expression, Hawaiʻi

## Abstract

**Background:**

A striking aspect of evolution is that it often converges on similar trajectories. Evolutionary convergence can occur in deep time or over short time scales, and is associated with the imposition of similar selective pressures. Repeated convergent events provide a framework to infer the genetic basis of adaptive traits. The current study examines the genetic basis of secondary web loss within web-building spiders (Araneoidea). Specifically, we use a lineage of spiders in the genus *Tetragnatha* (Tetragnathidae) that has diverged into two clades associated with the relatively recent (5 mya) colonization of, and subsequent adaptive radiation within, the Hawaiian Islands. One clade has adopted a cursorial lifestyle, and the other has retained the ancestral behavior of capturing prey with sticky orb webs. We explore how these behavioral phenotypes are reflected in the morphology of the spinning apparatus and internal silk glands, and the expression of silk genes. Several sister families to the Tetragnathidae have undergone similar web loss, so we also ask whether convergent patterns of selection can be detected in these lineages.

**Results:**

The cursorial clade has lost spigots associated with the sticky spiral of the orb web. This appears to have been accompanied by loss of silk glands themselves. We generated phylogenies of silk proteins (spidroins), which showed that the transcriptomes of cursorial *Tetragnatha* contain all major spidroins except for flagelliform. We also found an uncharacterized spidroin that has higher expression in cursorial species. We found evidence for convergent selection acting on this spidroin, as well as genes involved in protein metabolism, in the cursorial *Tetragnatha* and divergent cursorial lineages in the families Malkaridae and Mimetidae.

**Conclusions:**

Our results provide strong evidence that independent web loss events and the associated adoption of a cursorial lifestyle are based on similar genetic mechanisms. Many genes we identified as having evolved convergently are associated with protein synthesis, degradation, and processing, which are processes that play important roles in silk production. This study demonstrates, in the case of independent evolution of web loss, that similar selective pressures act on many of the same genes to produce the same phenotypes and behaviors.

**Supplementary Information:**

The online version contains supplementary material available at 10.1186/s12862-021-01779-9.

## Background

Convergent evolution occurs across many scales of biological complexity, from molecules to behavior, and across phylogenetic scales, from species to genera and families [1]. Some of the best examples of convergence are found in archipelago settings, where similar sets of ecological forms have evolved mostly independently on the constituent islands, e.g. Anolis lizards in the Caribbean [2] and Hawaiian spiders [3]. Convergence also produces similar ecological forms at much deeper phylogenetic scales, with well known examples in Australian marsupials that have convergent counterparts among placental mammals in other parts of the world [4]. In both these situations, geographic isolation leads to the convergence of sets of species that look similar when comparing across different geographical settings (e.g. Australian thylacines and American canids), but are most closely related to species that are divergent in ecology and live in the same area. In other situations, convergence is imposed by similar selective pressures across transitions to marine [5] or high altitude [6] environments, or with the adoption of phenotypes such as social behavior [7] and echolocation [8]. In these cases, convergence is associated with repeated gains of a suite of traits. In other environments convergence can lead to trait regression, or the tendency of organisms to lose traits when released from selection for those traits, particularly when the traits are costly. For example, birds and insects frequently become flightless on islands [9], and cave organisms tend to lose eyes and pigmentation [10]. Loss of structures may be associated with neutral evolution—the accumulation of random mutations in genes associated with the regressed trait—or natural selection, which can be positive (e.g. energy gain as a result of loss of the trait [11]) or negative (the trait is deleterious). Therefore, a critical step in understanding the evolution of convergent traits, regressive or otherwise, is not only linking behavior to morphology, but linking both to the molecular basis for a given trait [12]. In particular, in cases where similar phenotypes are displayed by distantly related taxa, to what extent can alternative genetic pathways operate to create similar suites of traits [13], and at what point in the formation of the phenotype [1]?

The current study examines the phenomenon of web loss in a lineage of orb–web spiders in order to understand the relationship between behavior, morphology, and the genomic basis of the web-spinning trait. Spiders provide an ideal system for linking functional morphology (spinneret spigots and silk glands) and behavior (web building) to specific changes in the genome in order to understand how selection operates on complex traits (e.g. [14, 15]), building upon existing knowledge of the genetic basis of silk production. Spider silk fibers are primarily made up of proteins called spidroins—a contraction of “spider fibroin” [16]. Spidroins are typically large proteins with a unique structure consisting of long regions of tandem repeats flanked by non-repetitive amino and carboxyl (N- and C-) terminal domains [16, 17]. Studies have shown a clear correlation between expression at the transcriptome level and the demonstration of web spinning behaviors [18]. Moreover, adoption of a cursorial lifestyle can lead to changes in silk expression, such as increased production of major ampullate (dragline) silk [19].

The evolution of spider silk has received considerable attention [17, 20–23]. More derived spiders possess multiple distinct silk glands, which are serially homologous. These appear to have diversified through duplication, attaining different properties over their evolutionary histories [23]. Each silk gland comprises a sac, which connects through a duct of variable length and diameter to an excretory duct or spigot, located on a spinneret [24]. Within the sac of each gland, the spider synthesizes a fiber or glue with specific mechanical properties that are the result of gland-specific gene expression [16]. Within the superfamily Araneoidea, adult females can have up to seven types of silk glands—piriform (attachment cement), aciniform (wrapping), tubuliform (egg sac covering), major ampullate (dragline), minor ampullate (temporary spiral), aggregate (sticky glue) and flagelliform (capture spiral); the silk associated with each type of gland is extruded through a corresponding spigot [24, 25]. The spigots associated with spinning these different silks have well-defined morphologies and placement within the spinning fields on the spider spinnerets. Thus, the anterior lateral spinnerets (ALS) contain the major ampullate spigot and numerous piriform spigots; the posterior medial spinnerets (PMS) contain the minor ampullate spigot, a tubuliform spigot, and numerous aciniform spigots; and the posterior lateral spinnerets (PLS) contain another tubuliform spigot, numerous aciniform spigots, two aggregate spigots, and a single flagelliform spigot [25, 26]. The spinning of sticky capture silk is associated with a morphological structure located on each PLS: the “aggregate/flagelliform triad” consisting of two aggregate spigots and one flagelliform spigot [26]. This triad is a key synapomorphy of spiders in the superfamily Araneoidea [26, 27]. The flagelliform gland, and associated single spigot, produces a core line while the silk from the aggregate gland spigots coats this line with glue; the ducts of the aggregate and flagelliform glands are also physically attached [28]. In structure, the flagelliform glands consist of a long duct with a cylindrical proximal portion, similar to the ampullate glands, and are variable in size, smaller in the Linyphiidae and Theridiidae relative to the Araneidae, each of these families being closely related to Tetragnathidae [24]. The two pairs of aggregate glands are unusual—large, multilobed structures that appear to have been derived from coalescence of a number of smaller glands [24].

Although the sticky capture threads, and associated well-developed aggregate and flagelliform triad, are characteristic of the Araneoidea, the use of the sticky capture threads is frequently lost, and with it the aggregate and flagelliform triad. In most araneoids, adult males adopt a vagrant lifestyle and the triplet is lost [28] or vestigial [29, 30] in the final instar. Moreover, there are multiple independent cases where entire lineages have abandoned web building, and these cases are also accompanied by loss of the aggregate/flagelliform triad [26, 31–35]. Thus, it appears that convergent trait regression, with repeated loss of the sticky spiral triad of spinneret spigots, is tied to the adoption of a cursorial lifestyle. However, it is not clear whether these convergent phenotypic changes are reflected at the molecular level, and hence the relationship between genotype and phenotype remains unknown. In particular, given that the trait is convergent, are the morphological and behavioral similarities the result of similar changes at the molecular level, and are the selective pressures also acting in a similar way?

The current study examines convergent trait regression associated with the adoption of a cursorial lifestyle within the family Tetragnathidae, and asks whether changes in selection act on the same genes as in the related families of Mimetidae, Malkaridae, and Arkyidae, which are characterized by a similar adoption of a cursorial lifestyle and the absence of the aggregate/flagelliform triad [33]. The family Tetragnathidae—and presumably the ancestors of Mimetidae, Malkaridae, and Arkyidae—share many behavioral and morphological synapomorphies with other members of Araneoidea, including the presence of the aggregate/flagelliform triad for producing viscid sticky silk, and the movements involved in orb web construction [26]. However, partial loss of the triad has been documented in multiple lineages within the Tetragnathidae. The current study focuses on another tetragnathid lineage, a large species radiation in Hawaiʻi within the genus *Tetragnatha* [36]. This lineage appears to be monophyletic, with early divergence, ca. 5 million years ago [36], into two major clades. One of these clades has retained the web-spinning behavior that characterizes the genus, while the other, known as the “Spiny Leg” clade, has abandoned web building and adopted a cursorial lifestyle [37]. The parallel radiation of these two lineages provides an ideal opportunity to compare selective pressures acting on each clade. We examine how changes in the behavior of the Spiny Leg lineage relative to web spinners are reflected in (1) the morphology of the silk spinning apparatus, in particular the aggregate/flagelliform triad; (2) the presence of the silk glands themselves; and (3) changes in gene expression. Finally, we ask (4) whether natural selection has acted convergently in Spiny Leg *Tetragnatha* and in the clades Mimetidae, Malkaridae, and Arkyidae, where web loss has occurred independently. We sequenced six new transcriptomes and conducted a transcriptomic analysis between web building and non-web building members of the genus *Tetragnatha*, then tested for evidence of selection associated with web loss and the associated adoption of a cursorial lifestyle across the *Tetragnatha* phylogeny. We also tested for convergent selection using available transcriptomes from Mimetidae, Malkaridae, and Arkyidae.

## Results

### Morphology of spinneret spigots and silk glands

**Fig. 1:**
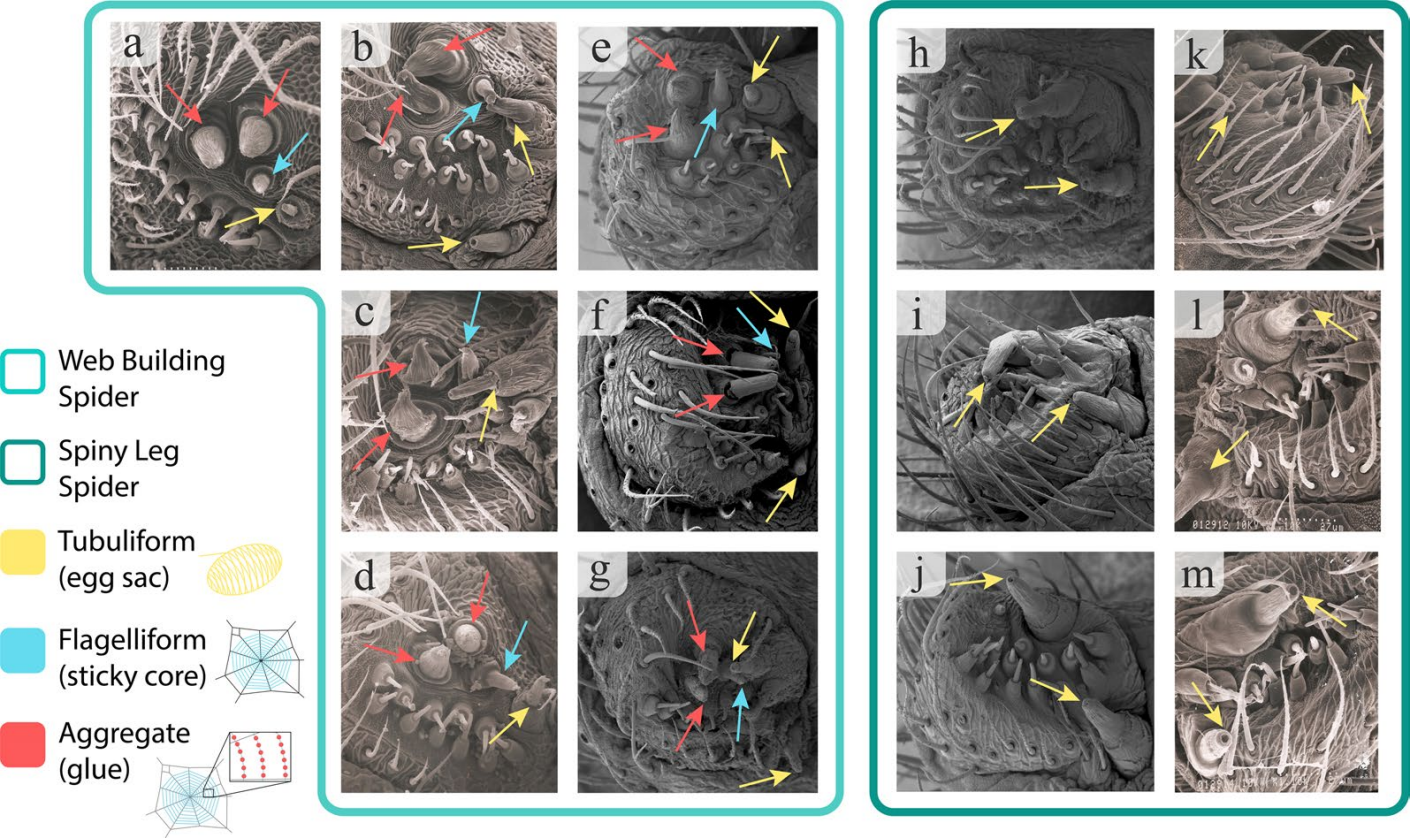
Scanning Electron Microscopy Images of the posterior lateral spinnerets. Web builders (7) shown on the left: **a** *Tetragnatha acuta*, **b** *T. trituberculata*, **c** *T. hawaiensis*, **d** *T. eurychasma* (photos taken with protocol used in 1990), **e** *T. sp.* “Little wave”, **f** *T. stelarobusta*, and **g** *T. sp.* “Long-clawed legs” (photos taken with protocol used in 2016). Spiny Leg clade (6) on the right: **h** *T. kauaiensis*, **i** *T. quasimodo*, **j** *T. macracantha* (photos taken with protocol used in 2016); and **k** and **l** *T. waikamoi* (2 individuals) and **m** *T. quasimodo* (photos taken with protocol used in 1990). Yellow arrows: tubuliform spigots; blue arrows: flagelliform spigots; green arrows: aggregate spigots. Unlabelled spigots are aciniform

**Fig. 2:**
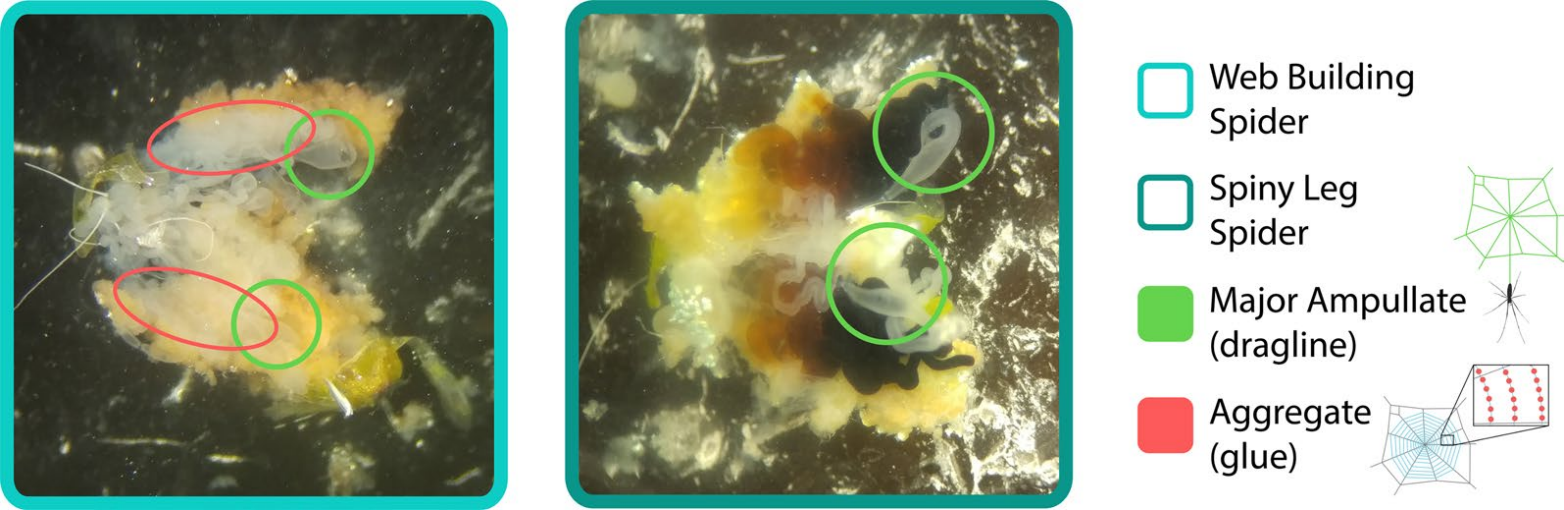
Representative photographs of dissected abdomens showing silk glands. Aggregate glands (red) are large, multilobed, whitish structures that are only visible in web-building species, shown on the left. Major ampullate glands (green) are whitish but smaller, and have a distinctive duct shape that is easy to recognize. For simplicity, other glands are not labelled (or are too small to see). Yellowish lumps are adipose tissue. Left: Web builder, *T. paludicola*. Right: Spiny leg, *T. brevignatha*

The most remarkable feature of the posterior lateral spinnerets is the very obvious presence of the aggregate and flagelliform spigots in all seven species of web-spinners (Fig. 1); these spigots form a triad associated with the production of sticky silk used in web-building. However, the four species in the Spiny Leg clade all demonstrate a complete absence of the aggregate and flagelliform spigots. Dissections were perfomed on three web-building (*T. stelarobusta, T. filiciphilia, T. paludicola*) and three Spiny Leg species (*T. brevignatha, T. quasimodo, T. waikamoi*); aggregate glands were easily identified as large, multilobed, semi-transparent white structures in all web-builders, and were found in none of the three Spiny Leg species (Fig. 2). Flagelliform glands are much smaller and more difficult to identify [24]. For this reason, we are not able to say with confidence that flagelliform glands are definitively absent from Spiny Leg spiders; however, aggregate glands have clearly been lost.

### Transcriptome assemblies

We have assembled the first protein-coding transcriptomes of six species of Hawaiian *Tetragnatha* spiders. We generated twelve RNA-seq libraries from the dissected abdomens of adult female spiders (two individuals per species); library and assembly statistics are given in Additional file 1. Sequencing depth ranged between 20.6 and 36.5 million paired-end 100 bp reads. Quality filtering removed an average of 6.57% of raw reads. After trimming, libraries contained between 19 and 33 million paired-end reads per individual, or between 43 and 62 million reads per species, with fragment lengths between 50 and 101 bp. De novo assemblies made with Trinity contained between 101,722 and 122,506 transcripts with a minimum contig length of 201 bp. The GC content of each species assembly was ~ 37%. All libraries had greater than 80% of reads map as proper pairs to their respective assembly using Bowtie2. The six assemblies recovered between 96.4 and 97.8% of the BUSCO odb09 set of 1066 core arthropod genes, which is at the high end for de novo transcriptome assemblies and indicates good coverage of the protein-coding transcriptomes. After predicting coding sequences using TransDecoder and removing redundant peptides (at 100% identity) with CD-HIT, the assemblies contained between 23,774 and 28,202 nonredundant peptides.

### Spidroin phylogenetic analysis

**Fig. 3:**
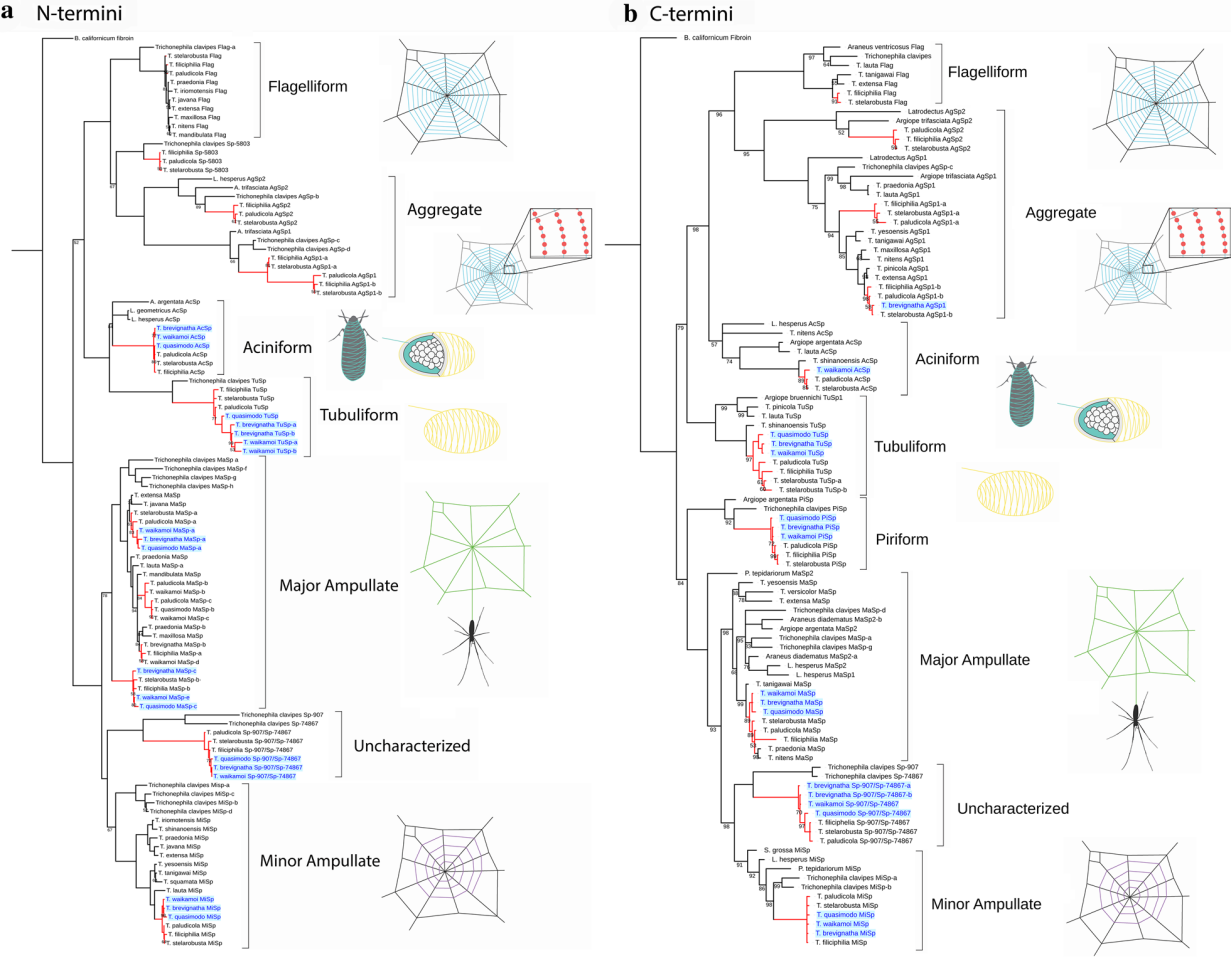
Bayesian consensus trees of terminal domains. **a** N-terminal sequences. **b** C-terminal sequences. Branch labels show posterior probabilities; where not labelled, support values are 100%. Branches leading to species sequenced in this study are colored red, and Spiny Leg species are labelled with blue text. Aggregate spidroins are numbered according to the the numbering system of [73]. Letters are used to distinguish multiple copies of spidroins in *Tetragnatha* (e.g. MaSp-a, MaSp-b). Where abbreviated, genus names are: A.: *Argiope*; T.: *Tetragnatha*; L.:Latrodectus; P.: Parasteatoda; S.: *Steatoda*. Trees produced using Mr. Bayes v3.2.7 and visualized with the iTOL web server

Full-length spidroins are difficult to assemble due to their unique structure, so we limited our analysis to N- and C-terminal domain sequences. We searched the predicted peptides for spidroin terminal domains using BLAST and constructed Bayesian consensus trees for both sets of termini (Fig. 3). We identified sequences belonging to the seven major spidroin clades (aciniform, aggregate, flagelliform, major ampullate, minor ampullate, piriform, and tubuliform), as well as three uncharacterized spidroins with homology to genes that had previously been identified only in the orb-weaving genus *Trichonephila* (Sp-5803, Sp-907/74867, and Sp-1339) [14, 38]. Phylogenetic conflict can occur between spidroin N- and C-termini, and N-termini generally contain more phylogenetic signal [21, 39]. Nonetheless, the only major topological difference between our two trees is that the N-terminal tree recovers a sister relationship between aciniform and tubuliform (as in [21]), whereas the C-terminal tree places aciniform sister to (flagelliform + aggregate). Note that with our data, we have no way of knowing which N- and C-terminal sequences belong to the same physical gene. None of the three Spiny Leg transcriptomes contained sequences belonging to the flagelliform or Sp-5803 clades. Hawaiian *Tetragnatha* appear to possess three aggregate spidroin genes, two of which are expressed in some Spiny Leg species (Additional file 2).

### Differential expression analysis and gene ontology enrichment

**Fig. 4:**
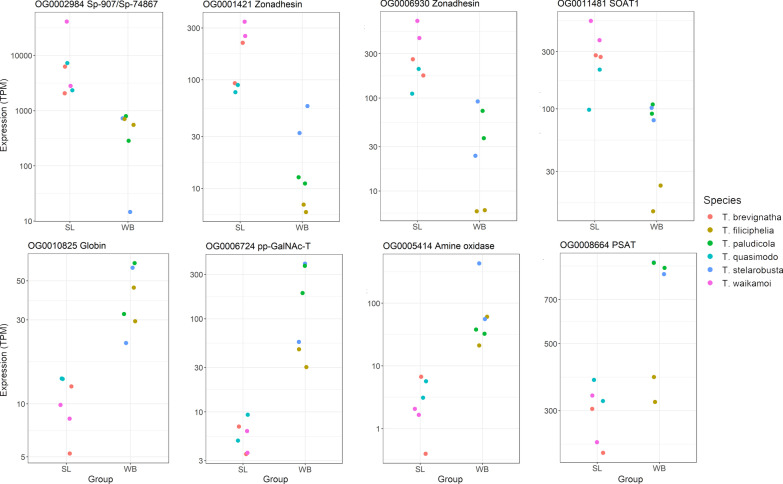
Relative expression of select genes. Counts have been normalized to average transcript size and library size, and are expressed as transcripts per million (TPM) on a log scale. SL: Spiny Legs; WB: web builders. SOAT1: Sterol O-Acyltransferase 1; pp-GalNAc-T: Polypeptide N-acetylgalactosaminyltransferase; PSAT: Phosphoserine aminotransferase

OrthoFinder assigned predicted proteins to 19,675 orthogroups, and PhyloTreePruner identified 7835 single-copy orthologs with genes in all six taxa, which were used for differential expression analysis between web builders and Spiny Legs. We identified 107 (1.4%) differentially expressed (DE) single-copy orthologs; of these, 49 were more highly expressed in Spiny Legs and 58 were more highly expressed in web builders. DE genes and their annotations are given in Additional file 3. Seventeen out of the 107 DE genes could not be annotated because they had no significant BLASTP hit against NCBI’s nr database (accessed November 23, 2020), or because the only hits were to hypothetical or uncharacterized proteins. One spidroin, Sp-907/Sp-74867, was more highly expressed in Spiny Legs. Many of the remaining DE genes (both up- and downregulated) belong to groups of genes previously identified as having silk gland-specific expression (in [18, 38, 40]), including proteases, transferases, and genes associated with cellular transport and secretion; the expression of some of these genes is shown in Fig. 4. Genes with higher expression in Spiny Legs include protease inhibitors such as zonadhesins, as well as transferases and transporter proteins. We observed reduced expression in Spiny Legs of some genes with aggregate gland-specific expression in other spiders, including an N-acetylgalactosaminyltransferase (pp-GalNAc-T) [41] and a globin protein [40]. Other genes with lower expression in Spiny Legs include an aminotransferase, a semaphorin membrane protein, and a cathepsin endopeptidase. Genes with higher expression in Spiny Legs were enriched for two gene ontology (GO) categories (FDR < 0.1): “chitin binding” and “transferring acyl groups other than amino-acyl groups”. Genes with higher expression in web builders were not enriched for any GO categories.

### Selection analyses

**Fig. 5:**
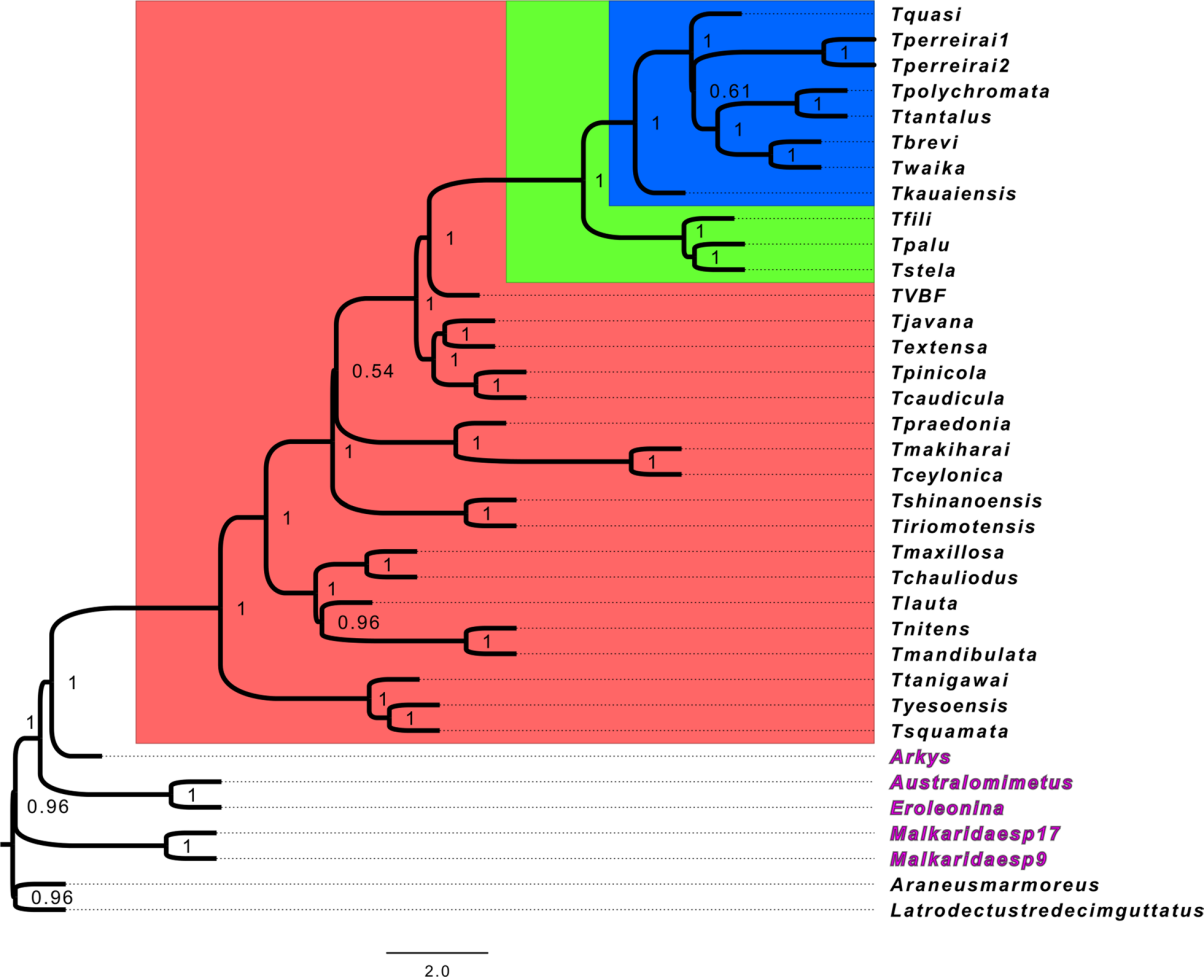
Species tree used for COATS branch tests, generated with ASTRAL. Red: *Tetragnatha* genus. Green and Blue: Hawaiian *Tetragnatha* lineage. Blue: Spiny Leg lineage. Branch support values indicate local posterior probabilities. Tree produced with ASTRAL v5.7.3

The first approach using the FUSTr [42] pipeline identified eight gene families corresponding to spidroins: of these, two contained sites showing evidence for pervasive positive selection, an aggregate spidroin and a major ampullate spidroin (Table 1). Neither family included sequences from Hawaiian *Tetragnatha*. Five of the remaining six spidroins included sequences from non-web building taxa and were further analyzed for selection along branches leading to those taxa. Three genes showed evidence for relaxed selection: an aggregate spidroin exhibited relaxed selection in the Spiny Leg *T. tantalus*, spidroin Sp-1339 exhibited relaxed selection in all Hawaiian *Tetragnatha* (web builders and Spiny Legs), and spidroin Sp-907/Sp-74867 exhibited relaxed selection in the non-web builders *T. waikamoi*, *T. brevignatha*, all analyzed species of Malkaridae, and *Australomimetus* sp. Spidroin Sp-907/Sp-74867 also showed evidence for episodic positive (diversifying) selection in Malkaridae. Finally, another aggregate sequence showed evidence for episodic positive selection in the Spiny Leg *T. polychromata*. For the second analysis using the COATS pipeline, the best-fit model involved positive selection along just the Spiny Leg branch for ten genes (Table 2; species tree used for COATS analysis shown in Fig. 5). These included ribosomal proteins and genes involved in protein ubiquitination and vesicle transport. For 24 genes, the best model involved selection in all non-web building taxa; these genes included mitochondrial enzymes, ATPases, proteasome components, and additional genes related to protein synthesis and ubiquitination (Table 2). None of these 34 genes overlapped with the set of genes that were differentially expressed between Spiny Legs and web builders.

**Table 1:** Results of spidroin gene family selection analyses

FUSTr family	Annotation	Pervasive selection (FUBAR)	Episodic selection (aBSREL)	Relaxed selection (RELAX)	Relax K
Family_5412	Sp-907/Sp-74867 (C-terminal)	NA	***Malkaridae sp. 17***	***Australomimetus sp., Malkaridae sp. 17, T. brevignatha,*** ***T. waikamoi***	K = 0.22
Family_18703	Sp-1339 (C-terminal)	NA	NA	***T. brevignatha, T. filiciphilia, T. paludicola, T. perreirai****, ****T. quasimodo, T. stelarobusta, T. waikamoi***	K = 0.31
Family_19435	AgSp (repetitive region)	NA	***T. polychromata***	NA	NA
Family_21837	AgSp (C-terminal)	NA	NA	***T. tantalus***	K = 0.09
Family_68763	MaSp (N-terminal)	15 residues	NA	NA	NA
Family_30334	AgSp (C-terminal)	3 residues	NA	NA	NA
Family_54136	AgSp (repetitive region)	NA	NA	NA	NA
Family_9974	AcSp (C-terminal)	NA	NA	NA	NA

For RELAX test, K parameter indicates degree of relaxation, with smaller values indicating greater relaxation in the test branches. AgSp, aggregate spidroin; MaSp, major ampullate spidroin; AcSp, Aciniform spidroin; Non-web building (cursorial) taxa are indicated in bold

**Table 2:** Results of COATS branch tests for positive selection

Locus	Length	Best model	Annotation
AM-TVBF-comp57563_c0_seq1.p1_RBHs	93	Non-web	26S proteasome non-ATPase regulatory subunit 14 (PSMD14)
TVBF-comp44613_c0_seq1.p1_RBHs	38	Non-web	ankyrin repeat domain-containing protein 27-like
AM-TVBF-comp45396_c0_seq1.p1_RBHs	240	Non-web	ATP synthase subunit b, mitochondrial
AM-TVBF-comp58985_c0_seq2.p1_RBHs	206	Non-web	ATP synthase subunit beta, mitochondrial
AM-TVBF-comp40348_c0_seq1.p1_RBHs	88	Non-web	Bystin-like (BYSL)
AM-TVBF-comp56406_c0_seq1.p1_RBHs	115	Non-web	Cilia- and flagella-associated protein 20
AM-TVBF-comp20801_c0_seq1.p1_RBHs	50	Non-web	E3 ubiquitin-protein ligase (MARCH1)
AM-TVBF-comp57815_c1_seq1.p1_RBHs	94	Non-web	E3 ubiquitin-protein ligase synoviolin (SYVN1)
AM-TVBF-comp53073_c0_seq1.p1_RBHs	101	Non-web	Electron transfer flavoprotein subunit beta (ETFB)
AM-TVBF-comp59149_c2_seq1.p1_RBHs	160	Non-web	MKI67 FHA domain-interacting nucleolar phosphoprotein-like
AM-TVBF-comp56762_c0_seq1.p1_RBHs	37	Non-web	N-alpha-acetyltransferase 35, NatC auxiliary subunit
AM-TVBF-comp49528_c2_seq1.p1_RBHs	122	Non-web	NEDD8-conjugating enzyme Ubc12 (UBE2M)
AM-TVBF-comp26055_c0_seq1.p1_RBHs	87	Non-web	Obg-like ATPase 1
AM-TVBF-comp36800_c0_seq1.p1_RBHs	138	Non-web	Peroxisomal biogenesis factor 19
AM-TVBF-comp53850_c1_seq1.p1_RBHs	91	Non-web	PRKCA-binding protein
TVBF-comp54710_c0_seq1.p1_RBHs	162	Non-web	Proteasome subunit alpha type-5 (PSMA5)
TVBF-comp148043_c0_seq1.p1_RBHs	149	Non-web	Putative ATP-dependent RNA helicase me31b
AM-TVBF-comp55490_c0_seq1.p1_RBHs	66	Non-web	Rab GDP dissociation inhibitor (GDI1)
TVBF-comp57571_c0_seq1.p1_RBHs	137	Non-web	Ras-related protein Rab-5
AM-TVBF-comp38265_c0_seq1.p1_RBHs\ml	102	Non-web	Ras-related protein Rap-2c
AM-TVBF-comp58242_c4_seq2.p1_RBHs	203	Non-web	Serine/threonine-protein kinase pim-3
AM-TVBF-comp56825_c0_seq1.p1_RBHs	115	Non-web	Synaptosomal-associated protein 29-like
AM-TVBF-comp58649_c1_seq1.p1_RBHs	115	Non-web	Transitional endoplasmic reticulum ATPase TER94
AM-TVBF-comp49097_c0_seq2.p1_RBHs	111	Non-web	V-type proton ATPase subunit E
AM-TVBF-comp40720_c0_seq1.p1_RBHs	102	Spiny Leg	39S ribosomal protein L10, mitochondrial (MRPL10)
AM-TVBF-comp57459_c0_seq1.p1_RBHs	264	Spiny Leg	60S acidic ribosomal protein P0 (RPLP0)
TVBF-comp58735_c0_seq1.p1_RBHs	196	Spiny Leg	DnaJ-like protein subfamily A member 1
TVBF-comp57965_c0_seq1.p1_RBHs	344	Spiny Leg	Elongation factor 2 (EEF2)
TVBF-comp49143_c0_seq2.p1_RBHs	131	Spiny Leg	NADH dehydrogenase [ubiquinone] iron-sulfur protein 3, mitochondrial
AM-TVBF-comp59471_c5_seq1.p1_RBHs	128	Spiny Leg	Splicing factor U2AF 50 kDa subunit
TVBF-comp49479_c0_seq1.p1_RBHs	158	Spiny Leg	Synaptobrevin (YKT6)
AM-TVBF-comp54231_c0_seq1.p1_RBHs	162	Spiny Leg	TATA-box-binding protein
AM-TVBF-comp54631_c0_seq1.p1_RBHs	72	Spiny Leg	Ubiquitin-conjugating enzyme E2 Q1 (UBE2Q1)
AM-TVBF-comp55223_c0_seq1.p1_RBHs	115	Spiny Leg	WD repeat-containing protein 44-like (WDR44)

Best model chosen by AIC. “Non-web” refers to a model in which selection acts on branches leading to “Spiny Leg” clade and to (Mimetidae + Malkaridae + Arkyidae). Spiny Leg refers to models where selection acts only on the Spiny Leg branch

## Discussion

The current study examined secondary loss of web building behavior within a radiation of Hawaiian spiders to determine the associations between behavior, morphology, and gene expression, specifically asking at which level selection appears to operate, and whether there is compensatory selection on other silks in association with the adoption of a cursorial lifestyle. We show that non-web building “Spiny Leg” *Tetragnatha* have lost aggregate and flagelliform spigots, as well as at least the aggregate glands. These behavioral and morphological changes are accompanied by shifts in gene expression and branch-specific selection. Intriguingly, we identify an important role for a novel spidroin in web loss, as this gene shows higher expression and shifts in selection in non-web builders. We also detect evidence for convergent evolution between Spiny Leg *Tetragnatha* and other non-web building lineages, not only in morphology (loss of aggregate and flagelliform spigots), but at the genomic level, with selection acting on some of the same genes across independent web loss events.

### Characterizing the transition to cursoriality in Spiny Leg *Tetragnatha*

#### Morphology of the silk spinning apparatus and associated silk glands

The web building Hawaiian *Tetragnatha* examined in this study clearly show a well-developed triad of the aggregate and flagelliform spigots. However, these structures appear to be completely absent in the Spiny Leg taxa examined (Fig. 1). The absence of these spigots parallels similar losses that have been documented for the Mimetidae, Malkaridae, and Arkyidae *s.l.* [35] (although mimetids may retain flagelliform and aggregate spigots as juveniles [43]). The lack of the aggregate/flagelliform triad is accompanied by an apparent absence of the associated aggregate and flagelliform glands in the Spiny Leg taxa—while the large aggregate glands are clearly lost, it is possible that the small flagelliform glands are merely highly reduced (Fig. 2). Similar shifts in morphology have been observed in other spider lineages in which the building of orb webs has been modified or lost. Notably, in *Cyrtophora* (Araneidae), loss of the triad has been associated with the loss of sticky silk in the production of the mesh of dry silk [32]; in the species *Drapetisca socialis* and *Neriene peltata* (Linyphiidae), a similar loss is associated with the ability to catch prey outside the web without using glue in the web [33]; and in the subfamily Argyrodinae (Theridiidae), loss of the triad spigots is linked to abandonment of web building behavior and the adoption of kleptoparasitic or free-living existence [34]. Reduction in the triad is also known in the families Synaphridae [29] and Micropholcommatidae [44], though the predatory behavior in these lineages is not known. Among the Tetragnathidae, the genus *Pachygnatha* has largely lost the behavior of web building for prey capture and adults have lost the triad [26], although juvenile *Pachygnatha* still spin an orb web [45]. Apart from the Spiny Leg species examined in this study, the loss of the triad has also been documented in other Hawaiian tetragnathids. The monospecific *Doryonychus raptor*, which captures its prey without a foraging web, shows no evidence of the triad even during the earliest developmental stages [31]; prey are captured, either in flight or from the substrate, by impalement with the claws, which occurs through a very rapid movement of one or more of the first two pairs of legs [46].

#### Changes in spidroin expression in Tetragnatha

The transcriptomes of web-building *Tetragnatha* contain every major type of spidroin that has been described for the superfamily Araneoidea, while the Spiny Leg transcriptomes contain all major spidroins except for flagelliform (Fig. 3). We caution that absence of evidence is not evidence of absence. However, given the high completeness of our assemblies, the fact that flagelliform transcripts are absent from six separate Spiny Leg transcriptomes, and the absence of flagelliform spigots, we view it as likely that flagelliform spidroins are not expressed at appreciable levels. It remains to be seen whether flagelliform spidroins are present in Spiny Leg genomes, or have become pseudogenes due to loss of function and a release from selective restraints. In contrast, one species sequenced in this study (*T. brevignatha*) as well as three previously-sequenced Spiny Leg *Tetragnatha* [47] do express aggregate spidroins, despite having lost aggregate silk glands (Fig. 3, Additional file 2). It is surprising that aggregate spidroins are expressed in species that cannot make aggregate silk. Spidroins are sometimes co-expressed in more than one silk gland [23, 48], and this could suggest that aggregate spidroins play a role in the production of other types of silk. Alternatively, it is likely that, because silk glands are derived from each other by serial homology, the ongoing transcription of aggregate spidroins is a remnant of past homology. This latter hypothesis is supported by the observation that aggregate transcripts in the Spiny Legs *T. brevignatha* and *T. waikamoi* were all expressed at quite low levels (< 1 TPM), whereas web builders expressed aggregate spidroins at high levels (> 100 TPM). Other studies have reported a similar phenomenon in male theridiid spiders, where loss of aggregate glands occurs upon reaching maturity, yet aggregate spidroins continue to be expressed [19]. Our selection analyses show that one aggregate spidroin appears to have experienced episodic diversifying selection in the Spiny Leg *T. polychromata* (Table 1), which suggests that it is performing some biological function. On the other hand, another aggregate spidroin has experienced relaxed selection in the Spiny Leg *T. tantalus*, which is consistent with a loss of function after the loss of aggregate silk production.

Spidroins generally fall into one of seven monophyletic groups corresponding to the seven silk glands [14]. However, the recent genome of the golden orb-weaver *Trichonephila clavipes* revealed several novel spidroins that do not fall into these well-established clades [14]. Our *Tetragnatha* transcriptomes contain homologs of some of these uncharacterized spidroins. Sp-5803 is primarily expressed in the flagelliform gland in *T. clavipes* [14, 40] and, like flagelliform spidroins, is absent from the Spiny Leg transcriptomes in this study, while being found in web builders (Fig. 3). Another uncharacterized spidroin, Sp-1339, shows evidence of relaxed selection in the entire Hawaiian *Tetragnatha* lineage (Table 1). A third spidroin, which forms a clade with *T. clavipes* Sp-907 and Sp-74867 (Fig. 3), has higher expression in Spiny Legs and has experienced relaxed selection both in Spiny Legs and in other non-web builders. We refer to this novel spidroin as Sp-907/Sp-74687. A previous study of major ampullate spidroins (MaSp) identified an expansion of MaSp-like sequences in *Tetragnatha*, including a “divergent spidroin variant” that lacked certain conserved residues [49]. These MaSp-like sequences (subtype F in [49]) are Sp-907/Sp-74687, having almost identical N-terminal domains (Additional file 4).

#### Transcriptional shifts of silk gland-associated genes accompany loss of web-building

Although spidroins make up the silk fibers themselves, many other genes play roles in silk production and have silk gland-specific expression. Previous studies have found that both peptidases and peptidase inhibitors are enriched among silk gland-specific genes. Clarke et al. suggest that this is because silk proteins that are successfully exported from cells need to be protected from degradation by peptidase inhibitors, whereas proteins that fail to properly assemble and export need to be degraded by peptidases [18]. Our results are consistent with this model, as both peptidases (e.g. a cathepisin and elastase) and peptidase inhibitors such as zonadhesins have shifted in expression between web builders and Spiny Legs. Interestingly, zonadhesins have been detected in the silk of the greater wax moth, which is not homologous to spider silk [50]. Silk gland-specific transcripts also include genes related to oxygen metabolism, at least in black widow spiders [18, 51]. In the current study, we observe the downregulation of an amine oxidase and a monooxygenase in Spiny Legs, but the upregulation of a glutaredoxin and a dual oxidase. We also observed that Spiny Legs downregulate two genes that are highly expressed in the major ampullate glands of black widow spiders, a phosphoserine aminotransferase and a gamma-glutamyltransferase, the latter potentially involved in redox regulation in silk glands [48].

Genes with higher expression in Spiny Legs are enriched for functions related to chitin binding. While chitin serves primarily as a major structural component of arthropod bodies, forming the cuticle or exoskeleton, it is also found within the walls and ducts of silk glands, such as the major ampullate silk glands of *Trichonephila clavipes* and even the non-homologous silk glands of the silkworm [52]. Some spidroins also possess chitin-binding domains [53]. The close relationship between chitin and silk suggests that the changes in gene expression observed here may reflect morphological changes accompanying the loss of internal silk glands and external spigots. It is unclear why Spiny Legs have increased expression of chitin-binding genes. However, the loss of aggregate and flagelliform silk could be accompanied by increased expression of other types of silk such as dragline [19], leading to higher expression of some silk-related genes.

Consistent with the loss of aggregate glands, Spiny Legs exhibited reduced expression of genes with aggregate gland-specific expression in other spiders, including a pp-GalNAc-T, a type of transferase that participates in O-glycosylation [54]. O-glycosylation contributes to the adhesive qualities of aggregate glue [55], and pp-GalNAc-T is highly expressed in the aggregate glands of the orb-weaver *Argiope trifasciata* [41]. The reduced expression of this gene in Spiny Legs accompanied by their loss of aggregate glands suggests that this gene could play a similar role in aggregate silk production in tetragnathids. Spiny Legs also have reduced expression of a globin protein, a type of protein specifically expressed in the aggregate glands of *Trichonephila clavipes* [40]. Spiny Legs have also downregulated other genes identified in the aggregate and flagelliform glands of *Trichonephila clavipes* [40], including an aminopeptidase, a serine/threonine protein kinase, and an acyl-CoA dehydrogenase. However, further work is needed to understand gland-specific patterns of gene expression in *Tetragnatha*.

### Convergent web loss is associated with selection on protein and energy metabolism

Branch tests of single-copy orthologs identified targets of selection along branches where independent web loss events have occurred in *Tetragnatha* and related lineages (species shown in Fig. 5). Many of the same genes, including ubiquitin ligases and proteasome components, appear to be targets of selection in both Spiny Legs and other non-web building families (Table 2). Other genes in this category include pim-3, a kinase that regulates protein synthesis [56], and BYSL, which mediates ribosome biogenesis [57]. This suggests that the loss of web silk is accompanied by convergent selection on protein synthesis, processing, and degradation machinery in both Spiny Leg *Tetragnatha* and other non-web building araneoid spiders. Since silk is a proteinaceous material, changes in the silk needs of a species may require changes in genes regulating protein metabolism. There is an interesting parallel in silkworms, where QTL analysis identified polymorphism in a ribosomal subunit associated with silk synthesis [58]. Another interesting category of genes with convergent selection is related to energy metabolism, including both ATP synthase and ATPases. There may be an association between large-scale changes in silk production and changes in cellular metabolism. Other genes, including ribosomal components and genes related to vesicle transport, are targets of selection only in Spiny Leg *Tetragnatha*, suggesting that lineage-specific differences are also important. Still, it is notable that even in cases when selection has not acted on the same gene, it acts on genes with functions related to protein metabolism.

#### Role for a novel spidroin in web loss

We have identified an as-yet uncharacterized spidroin, Sp-907/Sp-74867, that shows evidence for convergent relaxed selection in Spiny Leg *Tetragnatha* and the non-web builders Malkaridae sp. 17 and *Australomimetus* sp*.*; this gene has also experienced episodic diversifying selection in Malkaridae sp. 17 (Table 1). Relaxed selection can be caused not only by loss of function, but also by a change in functional constraints upon adaptation to a new ecological niche, or a reduction in effective population size [59]. Since we also observed increased expression of Sp-907/Sp-74867 in Spiny Legs, we suggest a role for this spidroin in the transition to a cursorial lifestyle. The function of Sp-907/Sp-74867 is unclear; however, in *Trichonephila clavipes*, expression of Sp-74867 correlates with major ampullate (dragline) spidroin expression [14]. Correa-Garwhal et al. found that male *Steatoda grossa*, which are cursorial, have higher expression of major ampullate spidroins than females, and suggest that major ampullate silk has an increased or different role in cursorial males that could be due to differences in dragline silk [19]. Sp-907/Sp-74867 expression might also be related to changes in dragline silk related to the transition to cursoriality. Future work localizing Sp-907/Sp-74867 to particular silk glands would help clarify its function, and it may also be interesting to examine differences in the structural composition of silks from web building and non-web building taxa.

It is important to note that the genes identified in this study are not exhaustive. Our gene expression analysis is limited by small sample sizes, meaning that we only have power to detect the largest changes in expression. Moreover, RNA-seq data can inflate expression estimates of spidroins when reads derived from repetitive regions map to fragments containing portions of the repetitive region [60]. Our selection analyses are constrained by the relative incompleteness of some transcriptomes, which limits the number of single-copy orthologs that we can recover. The transcriptomes sequenced in this study are quite complete, but other assemblies were generated with different goals where completeness was less important [47, 61]. This type of analysis also neglects the roles of gene loss and duplication in character evolution. Finally, transcriptomic data are rarely able to reconstruct full-length spidroins, which means that we cannot match N- and C-terminal sequences. The sequences in our phylogenetic and selection analyses correspond to internal, N-, or C-terminal fragments of full-length spidroins, which may have experienced different selection regimes. Nonetheless, our complementary analyses provide a clearer picture of the types of genes involved in web loss and of the distribution of spidroins in non-web building *Tetragnatha*. In particular, protein synthesis and degradation machinery appear to be convergent targets of selection when capture webs are lost.

### Future directions

The loss of web building behavior has occurred independently at many different levels: within the genus *Tetragnatha*, between genera within the Tetragnathidae, and between the related families shown in this study. This system therefore provides an interesting opportunity to compare genomic shifts across phylogenetic scales. Intriguingly, web loss within *Tetragnatha* is not only associated with loss of the aggregate/flagelliform triad, but also with a marked ecological shift into forest habitats, the display of bright green coloration with red color polymorphism, and the development of greatly elongated macrosetae (“spines”) on the forelegs [46]. The American *T. viridis* is a bright green forest dweller that has abandoned web building and has elongated spines on the legs [62]; *T. laqueata* from the Bonin Islands is also a spiny green forest dweller, as are *T. squamata* from Japan, *T. tanigawai* from the Ryukus [63], and *T. viridorufa* from India [64]. Future work comparing the genomic basis for these suites of convergent traits will provide insights into the interplay between genotype and phenotype that allows these characters to evolve together.

## Conclusions

Phenotypic convergence has been broadly defined as the independent evolution of similar phenotypes. There are numerous examples of convergence, though only a small fraction of studies have examined whether convergent events are based on shared genetic mechanisms [12]. Our study is the first to systematically characterize silk genes in Spiny Leg *Tetragnatha*, and determine associations with convergent adaptation of a cursorial lifestyle. Web loss is associated with a series of morphological, ecological, and behavioral traits, and we show that web loss is accompanied by shifts in the expression of silk-associated genes within *Tetragnatha*. Our results further show that selection has acted on many of the same protein-coding genes in independent lineages that have lost webs and shifted to a cursorial lifestyle.

## Methods

### Morphology of spinneret spigots and silk glands

Scanning electron microscopy was conducted on the spinnerets of single individual females of seven web spinners: *Tetragnatha acuta*, *T. trituberculata*, *T. hawaiensis*, *T. eurychasma*, *T. sp.* “Little wave”, *T. stelarobusta*, and *T. sp.* “Long-clawed legs”; and four species in the Spiny Leg clade: *T. kauaiensis*, *T. quasimodo* (two individuals), *T. macracantha*, and *T. waikamoi* (two individuals). These are shown in Fig. 1. Specimens were imaged either in 1990 or 2016, following slightly different protocols. In the early study (1990), live spiders were boiled in water for 40 s in order to cause the spinneret spigots to evert. In more recent work (2016), dead specimens preserved in 70% ethanol were used; spinnerets were everted by pinching the abdominal tissue at their base with fine-tipped forceps. The spinnerets were then cut from the body and placed in plastic capsules with the central portion removed and nylon mesh placed inside the capsule (to allow exchange of alcohol and CO_2_, while retaining the specimen). Filled capsules were put through an alcohol series (70%, 85%, 95% and pure ethanol), then dried with an Autosamori-810 (in 1990) or a Bal-Tec CPD 030 (in 2016) critical point dryer. The structures were then removed from the capsules, mounted on stubs and sputtered with gold, and then viewed using a Hitachi S-800 (in 1990) or a Philips XL-30 ESEM (in 2016) scanning electron microscope.

### Animal collection for RNA extraction

We collected six species of *Tetragnatha* on Maui in June and September 2017: the web-builders *T. stelarobusta, T. filiciphilia,* and *T. paludicola*, and the Spiny Legs *T. waikamoi, T. brevignatha,* and *T. quasimodo*. We sequenced total RNA from two individuals of each species, for a total of twelve sequence libraries. All individuals were females and sexually mature at the time of extraction. The spiders were kept in the lab in Berkeley, California until they were extracted in November and December of that year. Animals were maintained under a common temperature, humidity, and light regime and fed at the same time each day. Extractions were performed on two days, at the same time each day. Live spiders were anesthetized with carbon dioxide and their abdomens were severed at the narrowest point of connection with the cephalothorax. Silk glands were dissected out as one mass in the presence of SSC buffer and preserved in RNAlater (procedure adapted from [65]). Due to the small size of the abdomens (approximately 5 mm in length), not all skin and abdominal tissue was excised. During dissections, each species’ complement of silk glands was observed. Excised tissues were homogenized and RNA extraction was performed using a Qiagen RNeasy Extraction Kit following the manufacturer’s protocol. RNA concentration and quality were verified for each sample using a Qubit fluorometer and a NanoDrop spectrophotometer. We also included a set of *Tetragnatha* spiders that were collected from different areas in Japan. These samples were all brought back to the lab alive (in less than a week), and were snap frozen with liquid nitrogen, then stored at −80˚C until they were subjected to RNA extraction. For collecting information and accession numbers, see Additional file 5.

### Library construction and sequencing

Library preparation was performed using a KAPA RNA Library Preparation Kit, following the manufacturer’s protocol. To summarize, poly-A mRNA selection was performed using magnetic oligo-dT beads. RNA was fragmented to the desired size (approx. 350–450 bp) by heating to 85 °C in fragmentation buffer. First-strand cDNA was synthesized with random primers, and second-strand cDNA was synthesized and marked with dUTP to preserve strandedness. Double-stranded cDNA libraries were A-tailed and ligated to dual-index Illumina adapters. The adapter-ligated libraries were amplified with 10 PCR cycles. Finally, library concentration and fragment size were assessed with a Bioanalyzer and Qubit fluorometer. Paired-end sequencing was performed on an Illumina HiSeq 4000 by the Vincent J. Coates Genomics Sequencing Laboratory at UC Berkeley. Sequencing for Japanese *Tetragnatha* (unpublished data) was performed as described previously in [66].

### De novo transcriptome assembly and filtering

Quality filtering was conducted with Trimmomatic v0.38 [67] using conservative parameters (LEADING:5 TRAILING:5 SLIDINGWINDOW:5:5 MINLEN:50). The six species transcriptomes were each assembled using Trinity v2.4.0 [68], with default parameters except for omitting in silico normalization (–no-normalize-reads). To evaluate assembly quality, we mapped trimmed reads back to their assembly using Bowtie v2.3.4 [69]. Assembly completeness was assessed with the arthropod set of BUSCO (Benchmarking Universal Single Copy Orthologs) v3.0.2 [70] (downloaded February 7, 2017).

### Protein prediction and annotation

We used TransDecoder v5.3.0 (http://transdecoder.github.io) to generate predicted open reading frames (ORF’s), with a minimum length of 100 amino acids. We subjected these candidate peptides to three homology searches: using DIAMOND v0.9.22.123 [71] (with the ‘–more-sensitive’ flag), we searched against NCBI's nonredundant nr protein database (downloaded on March 23, 2019), and against a custom database of N- and C-terminal spidroin sequences from unpublished *Tetragnatha* transcriptomes. We also used hmmscan v3.1b2 (available from hmmer.org) to search the Pfam protein database (downloaded February 22, 2019). TransDecoder retained the single best ORF for each transcript, and any predicted peptides with an e-value of less than 1e-10 against any database were also retained in the final set. We then used CD-HIT v4.7 [72] to merge peptides with 100% identity, to produce six non-redundant protein-coding transcriptomes. We also annotated single-copy orthologs used in the differential expression analysis (see below) with BLASTP v2.10.0 against the nr database (downloaded November 23, 2020), with an e-value cutoff of 1e−10, and these annotations are reported in Additional file 3.

### Orthogroup assignment

We used the program OrthoFinder v2.2.7 [73] to assign proteins to orthogroups and construct a rooted gene tree for each group. An orthogroup is a monophyletic group of genes descended from an ancestral gene in the last common ancestor of the species being analyzed, and can include both orthologs and paralogs. OrthoFinder also infers a species tree from the single-copy gene trees. We then used PhyloTreePruner v1.0 [74] to define a set of single-copy orthologs with one representative in each species. For gene trees with monophyletic species-specific gene duplications (in-paralogs), PhyloTreePruner retains only the longest paralog sequence. This approach is particularly relevant for transcriptomic data, as apparent in-paralogs are often alternative splice variants of the same gene.

### Transcript quantification and differential expression

Transcript abundance for each library was quantified using Salmon v0.13.1 [75]. Indices were constructed for the six transcriptomes with a k-mer size of 31, and mapping was performed with the following parameters: ‘–validateMappings –rangeFactorizationBins 4 –seqBias –gcBias’. We used the R package tximport v1.10.1 [76] to summarize transcript-level abundance to the orthogroup/gene level. In order to combine the data from different transcriptomes, we edited transcript names to append a species identifier to the Trinity contig name. For each library, we then added the transcript names for the other five species, with a count of zero. For example, a *T. waikamoi* counts file would include its count data for the *T. waikamoi* transcripts, and zero counts for all transcripts from the other five species. We provided a file relating transcript ID’s and single-copy orthogroups. This means that only transcripts belonging to one of the single-copy orthologs were retained for differential expression analysis, although reads were originally mapped to the entire transcriptome. Importantly, tximport corrects for differences in transcript length when summarizing to the gene level.

We used the R package DESeq2 v1.22.2 [77] to test for differential expression between the web building and Spiny Leg groups. The six individuals within each group (belonging to three species) were treated as replicates. We were able to compare gene expression across species due to the use of common garden conditions and use of a single life stage. Genes (single-copy orthologs) with log-fold change greater than or equal to 0.5 at an FDR of 0.05 were considered differentially expressed. We also estimated shrunken log-fold changes using the ‘lfcShrink()’ function with the method ‘apeglm’ and ‘lfcThreshold = 0.5′, and used these shrunken log-fold changes for gene ontology enrichment. The code for this analysis is available in Additional file 6.

### Gene ontology enrichment analysis

We retrieved GO annotations using Blast2GO v5.2.5 [78] on the output of the DIAMOND search against nr (with an e-value cutoff of 1e-10). This was done for each transcriptome, and orthogroups were annotated by combining the GO terms from all transcripts belonging to that orthogroup. Of the 7835 single-copy orthologs, 6648 received at least one GO annotation. GO enrichment was analyzed using the R package GO_MWU [79] on the shrunken log-fold changes, with default settings.

### Spidroin phylogeny

We aligned N- and C-terminal domain amino acid sequences with the M-Coffee web server [80]. Bayesian majority consensus trees were constructed using Mr. Bayes v3.2.7 [81] with a mixed amino acid model and a gamma rate distribution. Monte Carlo Markov Chain sampling was run for 5 million generations (or 1 million for the smaller aggregate spidroin tree), with four chains and a relative burn-in of 25%, for two independent runs. Trees were visualized on the iTOL web server [82] and rooted with a mygalomorph spidroin (*B. californicum* fibroin), or with flagelliform spidroins in the case of the aggregate spidroin tree.

### Selection analyses

These analyses set out to look at signatures of selection operating on the different silks. In particular, we tested for diversifying and relaxed selection associated with shifts from web building to cursorial lifestyle and with the loss of web building behavior. Moreover, we tested to see whether similar patterns of selection operated in different lineages where web loss has been documented. We included other published (from [47, 83]) and unpublished *Tetragnatha* transcriptomes, as well as transcriptomes from three other non-web building families (Arkyidae, Malkaridae, and Mimetidae) and two Araneoid web-building outgroups in the analyses (from [61]). We used several complementary approaches to test for selection in silk genes. First, we used a gene family clustering approach implemented in the FUSTr pipeline (Families Under Selection in Transcriptomes [42]), which clusters sequences from all available transcriptomes into putative gene families. These gene families do not necessarily contain sequences from all taxa, and given the structure of spidroins (discussed in the introduction) are likely to contain only fragments of a larger gene. As input, we used our six new *Tetragnatha* transcriptomes, 23 additional *Tetragnatha* transcriptomes corresponding to 22 species (*T. perreirai* represented twice) [47, 83], five non-web building spiders from other families from [61] (*Arkys* sp*.*, *Australomimetus* sp., *Ero leonina*, Malkaridae sp*.* 9, Malkaridae sp*.* 17), and two araneoid web building outgroup species from the same study (*Araneus marmoreus*, *Latrodectus tredecimguttatus*), for a total of 36 transcriptomes/35 species. FUStr translates sequences and predicts open reading frames using TransDecoder, assigns genes to homologous families using BLASTP and the clustering algorithm SiLix (v1.2.11) [84], generates multiple sequence alignments with MAFFT [85], and builds trees for each family using FastTree [86]. For each gene family with at least 15 sequences, we then conducted site tests for positive selection using FUBAR [87]. For gene families with BLAST hits to *Trichonpehila clavipes* spidroin sequences, we implemented branch tests using aBSREL [88] and RELAX [59], which test for diversifying and relaxed selection, respectively. FUBAR, aBSREL, and RELAX were implemented in HyPhy v2.5.14.

An additional strategy was used to test for selection in single-copy orthologs in the context of the species tree, using the COATS pipeline (unpublished, Brewer et al. in prep, https://hub.docker.com/r/michaelsbrewer/coats_test). Unlike FUSTr, COATS only considers single-copy orthologs present in all taxa, with histories that are consistent with the species tree. The species tree was generated from unrooted gene trees using ASTRAL v5.7.3 [89]. COATS generates orthologous gene sets using reciprocal best hits with BLASTP, which are then aligned with MAFFT [85] and codon alignments are generated with Pal2Nal [90]. Branch tests were then conducted using the codeml module of PAML [91]. We limited our analysis to orthologs consistent with the species tree, as evaluated with the approximately unbiased test in IQTREE v2.0 [92]. None of these single-copy orthologs were spidroins, likely because of the relative incompleteness of many of the transcriptomes included in the analysis. We tested selection in two scenarios, one in which selection acts on the branch leading to the Spiny Leg clade, and another in which selection acts on the branches leading to Spiny Legs and to the other non-web builders (essentially, testing for convergence).

## Supplementary Information

**Additional file 1.** Read and assembly statistics. a: Percent of trimmed reads mapping as proper pairs to the assembly using Bowtie2. b: Peptides predicted by TransDecoder. c: Peptides after clustering at 100% identity with CD-HIT.

**Additional file 2.** Bayesian consensus tree of aggregate spidroin C-terminal domains. Branch labels show posterior probabilities. Branches leading to species sequenced in this study are colored red, and Spiny Leg species are labelled with blue text. Tree produced using Mr. Bayes v3.2.7 and visualized with iTOL web server.

**Additional file 3.** List of differentially expressed genes and annotations. Includes top 3 BLASTP hits against NCBI’s nonredundant nr database (accessed November 23, 2020).

**Additional file 4.** N-terminal amino acid alignment of Sp-907/Sp-74867 sequences from Malay et al. [77]**.** The N-terminal domains of “MaSp-f” sequences in [77] are almost identical to Sp-907/Sp-74867, and differ from those of other MaSp sequences. Alignment generated with M-Coffee.

**Additional file 5.** Sample information. Includes collection information (locale and date) for individuals sequenced in this study, as well as GenBank accession numbers.

**Additional file 6.** R code used for differential expression analysis.

## Data Availability

The datasets supporting the conclusions of this article are available in the project GitLab repository, https://gitlab.com/Brevver/tetragnathasilks. Sequenced reads and assemblies are available on the Short Read Archive and the Transcriptome Shotgun Assembly (TSA) database, respectively (BioProject PRJNA650562). The TSA projects have been deposited at GenBank under the accessions GITM00000000, GITN00000000, GITO00000000, GITP00000000, GITQ00000000, and GITR00000000. The versions described in this paper are the first versions, numbered GITM01000000 and so on. All sample information and accession numbers are given in Additional file 5.

## Abbreviations

AcSp: Aciniform spidroin
AgSp: Aggregate spidroin
ALS: Anterior lateral spinnerets
ATP: Adenosine triphosphate
bp: Base pair
BUSCO: Benchmarking universal single copy orthologs
BYSL: Bystin-like
cDNA: Complementary deoxyribonucleic acid
C-terminus: Carboxyl terminus
DE: Differentially expressed
dUTP: Deoxyuridine triphosphate
FDR: False discovery rate
Flag: Flagelliform spidroin
GO: Gene ontology
MaSp: Major ampullate spidroin
MiSp: Minor ampullate spidroin
mya: Million years ago
NCBI: National Center for Biotechnology Information
N-terminus: Amino terminus
ORF: Open reading frame
PCR: Polymerase chain reaction
PiSp: Piriform spidroin
PLS: Posterior lateral spinnerets
PMS: Posterior medial spinnerets
pp-GalNAc-T: N-acetylgalactosaminyltransferase
QTL: Quantitative trait loci
RNA: Ribonucleic acid
SSC: Saline-sodium citrate
TPM: Transcripts per million
TuSp: Tubuliform spidroin
