# Supplementary figures and images for: Shifts in morphology, gene expression, and selection underlie web loss in Hawaiian Tetragnatha spiders
Source: BMC Ecol Evol. 2021 Mar 22;21:48. doi: 10.1186/s12862-021-01779-9 (PMC7983290; doi:10.1186/s12862-021-01779-9)

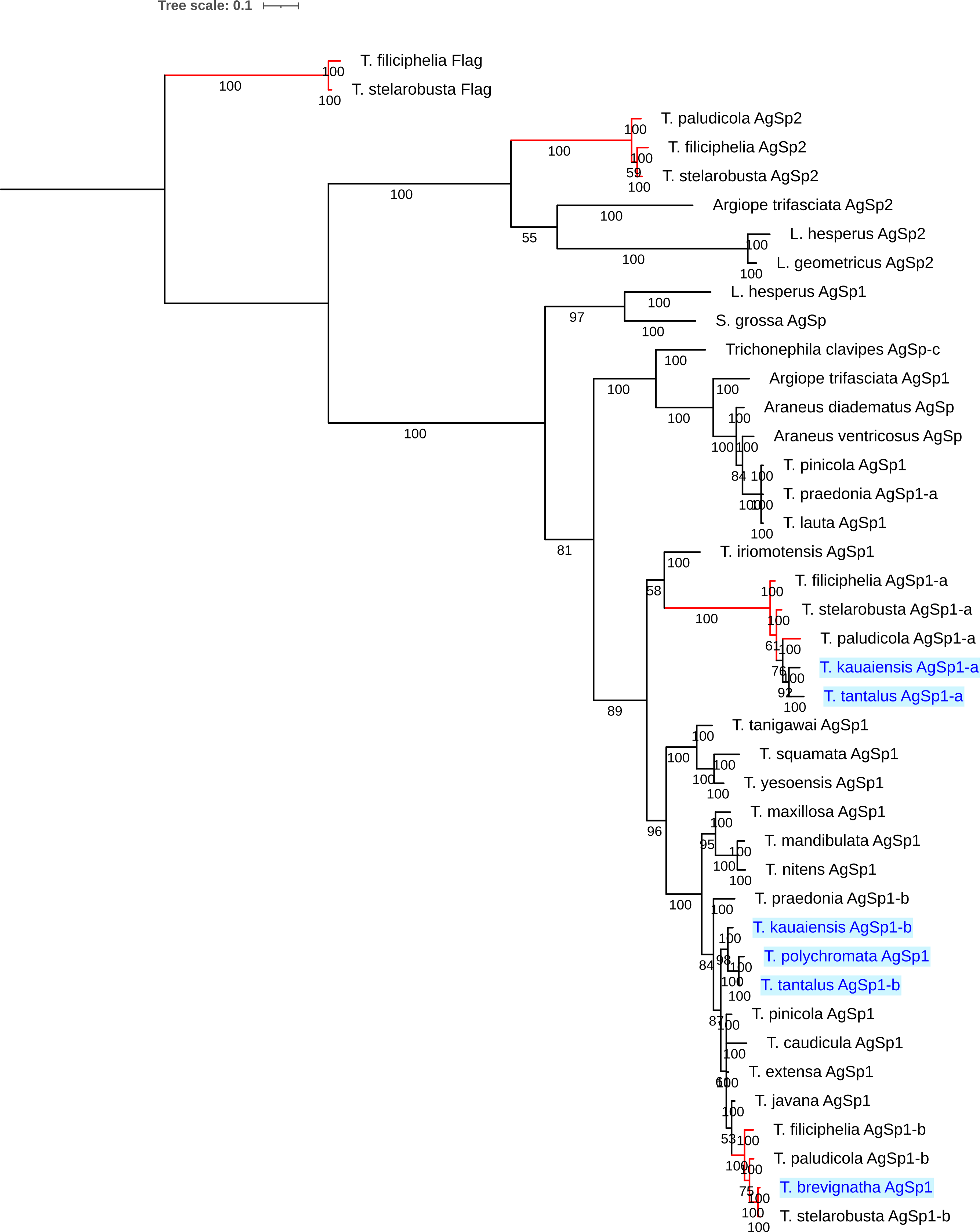

Supplement: Supplementary file 2 — Additional file 2. Bayesian consensus tree of aggregate spidroin C-terminal domains. Branch labels show posterior probabilities. Branches leading to species sequenced in this study are colored red, and Spiny Leg species are labelled with blue text. Tree produced using Mr. Bayes v3.2.7 and visualized with iTOL web server. [file 12862_2021_1779_MOESM2_ESM.png]
